# Microtubule Organization Is Essential for Maintaining Cellular Morphology and Function

**DOI:** 10.1155/2022/1623181

**Published:** 2022-03-07

**Authors:** Lijiang Huang, Yan Peng, Xuetao Tao, Xiaoxiao Ding, Rui Li, Yongsheng Jiang, Wei Zuo

**Affiliations:** ^1^The Affiliated Xiangshan Hospital of Wenzhou Medical University, No. 291 Donggu Road, Xiangshan County, Zhejiang 315000, China; ^2^Hangzhou Institute for Food and Drug Control, Hangzhou, Zhejiang, China; ^3^The Second Affiliated Hospital, Zhejiang University School of Medicine, Hangzhou, Zhejiang 310009, China; ^4^Department of Pharmacy, The People's Hospital of Beilun District, Ningbo, Zhejiang 315807, China; ^5^PCFM Lab, GD HPPC Lab, School of Chemistry, Sun Yat-sen University, Guangzhou 510275, China

## Abstract

Microtubules (MTs) are highly dynamic polymers essential for a wide range of cellular physiologies, such as acting as directional railways for intracellular transport and position, guiding chromosome segregation during cell division, and controlling cell polarity and morphogenesis. Evidence has established that maintaining microtubule (MT) stability in neurons is vital for fundamental cellular and developmental processes, such as neurodevelopment, degeneration, and regeneration. To fulfill these diverse functions, the nervous system employs an arsenal of microtubule-associated proteins (MAPs) to control MT organization and function. Subsequent studies have identified that the disruption of MT function in neurons is one of the most prevalent and important pathological features of traumatic nerve damage and neurodegenerative diseases and that this disruption manifests as a reduction in MT polymerization and concomitant deregulation of the MT cytoskeleton, as well as downregulation of microtubule-associated protein (MAP) expression. A variety of MT-targeting agents that reverse this pathological condition, which is regarded as a therapeutic opportunity to intervene the onset and development of these nervous system abnormalities, is currently under development. Here, we provide an overview of the MT-intrinsic organization process and how MAPs interact with the MT cytoskeleton to promote MT polymerization, stabilization, and bundling. We also highlight recent advances in MT-targeting therapeutic agents applied to various neurological disorders. Together, these findings increase our current understanding of the function and regulation of MT organization in nerve growth and regeneration.

## 1. Introduction

A multitude of cellular processes rely on the cytoskeleton, a filamentous scaffold of proteins that is essential for cell morphogenesis and division and intracellular transport. In eukaryotic cells, the cytoskeleton consists of three types of polymers: microtubules (MTs), actin filaments, and intermediate filaments. All three polymers provide a complex internal structure that maintains cellular homeostasis and fulfills different physiological functions. In general, actin filaments and MTs exhibit structural and regulatory interactions and such cytoskeletal crosstalk allows rapid intracellular reorganization, shape maintenance, and intracellular organelle movement [1, 2]. Additionally, all three polymers undergo dynamic assembly and disassembly through whole cellular processes [3]. This dynamic instability generates forces that drive changes in cell shape and motility [4].

In many cells, MTs are created through the spontaneous assembly of alpha/beta-tubulin dimers into polarized filaments, which allows them to act as structural scaffolds and signaling platforms for cellular behavior. During nervous system development, MTs are abundant in axons and dendrites and have a nearly uniform polarity orientation [5]. This oriented array allows the directional transport of cargoes to be properly orchestrated. Given the distinct polarity, organization, and posttranslational modifications, it is not surprising that microtubule (MT) organization and orientation are one of the most essential and earliest developmental differences between axons and dendrites [6]. In axons, MTs are uniformly orientated with their plus-ends towards the axon tip, whereas in dendrites, MTs are either oriented in the same manner or the opposite manner, depending on neuronal type and organism [7, 8]. Proper MT function as well as proper functioning of their assortment of interacting and regulatory proteins and regulatory pathways is particularly important in neurons. Abnormalities of the MT system in axons and dendrites, i.e., MT depolymerization, loss, or dysfunction or disorganized MT arrays, are a common insult during the pathogenesis of nerve traumatic disorders [9, 10].

Here, we summarize our current understanding of the basic biological features of MT organization within the cell, focusing on MT self-assembly and the direct participation of microtubule-associated proteins (MAPs) in MT nucleation, stabilization, and postmodification. We also discuss the current state of microtubule-stabilizing agents (MSAs), a new pharmacological intervention for treating central nervous system disorders, and address several microtubule-destabilizing agents (MDAs) as therapeutic strategies for suppressing cancer activity and vasculogenesis. Such diversified MT functions may provide us with new insights into MT-targeting therapies that mitigate structural and functional alterations linked to nervous system disorders.

## 2. MT Organization and Dynamic Regulation in Eukaryotic Cells

MTs are hollow cylindrical tubes consisting of repeating *α*-tubulin and *β*-tubulin heterodimers that play central roles in cellular morphogenesis, division, and development. The *α*-tubulin subunit is the minus-end in MT networks and exhibits slow growth rates and fast dissociation rates. The *β*-tubulin subunit is the plus-end and exhibits opposite growth and dissociation rates relative to the *α*-tubulin subunit. Within eukaryotic cells, tubulin dimers can form heterogeneous and dynamic protofilaments by aligning head to tail; approximately 13 protofilaments of MT are needed to form a hollow tube. During the process of MT polymerization, it usually exhibits dynamic instability, i.e., assembly and disassembly occur simultaneously, which is essential for normal functioning of the MT cytoskeleton [11]. The mechanism of such behavior is governed by the presence of two distinct states of GTP-GDP shafts at the *β*-tubulin end [12]. When tubulin dimers are free in solution, MT at the *β*-tubulin end is under the GTP-bound state and can be exchanged [13]. After incorporation of the tubulin dimers into the MT, *β*-tubulin hydrolyzes the GTP to GDP [14]. Thus, as long as subunits of GTP-bound *β*-tubulin form a GTP-tubulin cap at the plus-end [15], MTs can grow, but when GTP at the exchangeable site (E-site, located at the *α*/*β*-tubulin dimer of the plus-end) becomes hydrolyzed to GDP due to the GTPase activity of *β*-tubulin, the MT enters a state of shortening. With the help of the GTP-tubulin cap and E-site, MT polymerization and depolymerization occur primarily at the plus-ends.

**Figure 1:**
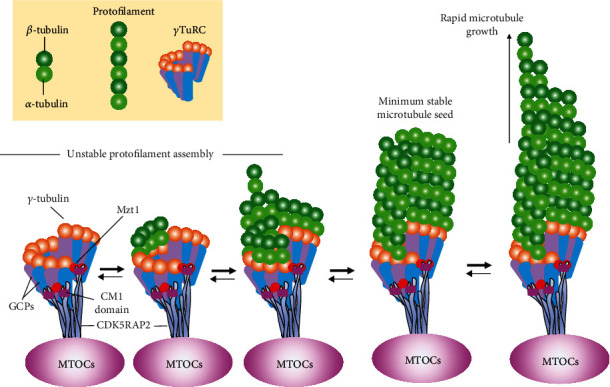
The process of *γ*-TuRC-mediated MT nucleation. The formation of the *γ*-TuRC ring structure and its interaction with *α*/*β*-tubulin dimers are described above. Briefly, the *γ*-TuRC complex can be recruited to various MTOCs via linking other accessory proteins to form a ring-like structure. Such structure allows the rapid growth of complex MT networks via *γ*-tubulin molecules interacting longitudinally with the MT minus-ends. It should be noted that MTs have the characteristic of dynamic instability, which allows MTs to spontaneously switch between assembly and disassembly phases. If there are sufficient tubulin dimers, MT polymerization can progress rapidly.

During the initial stage of MT formation (also termed MT nucleation, Figure 1), *α*/*β*-tubulin dimers need a template to guide assembly and elongation. This template is named the gamma-tubulin ring complex (*γ*-TuRC), which consists of numerous *γ*-tubulin molecules with various types of gamma-tubulin complex proteins (GCPs) [3]. GCPs at the N-terminal regions can interact directly with mitotic spindle organizing protein 1 (MZT1), a key regulator of the centromere structure [16]. The MZT1 protein is capable of binding to the N-terminal centrosomin motif 1 (CM1) domain of CDK5RAP2, a tethering protein that can recruit and bind *γ*-TuRC to diverse microtubule organizing centers (MTOCs), including the centrosome, Golgi apparatus, or mitotic chromatin [17, 18]. After forming the *γ*-TuRC ring structure, *γ*-tubulin molecules can anchor the minus-end of *α*/*β*-tubulin dimers to support a lateral association between *α*/*β*-tubulin dimers and polymerize into MTs with parallel orientations [19]. Thus, MT nucleation plays a crucial role in regulating MT self-assembly in various eukaryotic cells.

## 3. Types of MT Regulatory Proteins

MT stability, predominantly its mass and conformation, is controlled by the activities of several MAPs. They participate in a plethora of cellular processes, including cellular division, polarization, and intracellular transport, and can be categorized into MT-stabilizing proteins and MT-destabilizing proteins. The former group including MAP2, Tau, and Doublecortin (DCX), can interact with different MT-binding domains to bundle neighboring MTs [20]. MT-destabilizing proteins, on the other hand, perturb specific interaction nodes within the MT minus-end by accelerating the frequency of MT depolymerization. Different MAPs may regulate the stability and dynamics of MTs by creating different assembly patterns, either by altering the stability of lateral bonding tip extensions or altering the delivery of tubulin subunits to the tip [21, 22]. This regulation creates spatial and temporal patterns in the MT network within cells that are particularly important for cell morphogenesis, division, and physiology [23]. Although some MAPs remain poorly understood due to their high degree of homology, a huge number of mammalian MAPs have been characterized using proteomics and the construction of transgenic animal models [24, 25]. Here, we highlight some typical MAPs involved in their features and physiological functions as well as in the molecular mechanisms of stabilizing MTs.

MAP2, a microtubule-associated protein (MAP) family member, produces four different isoforms (MAP2A-D) according to alternative splicing of the same transcript. Generally, these four isoforms are highly expressed in differentiated neuronal dendrites, resulting in their utilization for labeling mature neurons. In addition, MAP2C and MAP2D are also widely distributed in glial cells. During the stages of neuronal development, MAP2 modulates MT-mediated cellular transport to participate in nucleation and stabilize MTs and bundling [26]. In particular, MAP2 is expressed in mature dendrites and is critically essential for protein synthesis and organelle biogenesis, as it controls cargo sorting at the predendritic filtering zone [27, 28].

Another MAP, Tau, also supports the stability and dynamics of the cytoskeleton but is mainly enriched in axons [29, 30]. While MTs and Tau share a conserved carboxy-terminal domain that can specifically interact under physiological conditions, the amino-terminal non-MT-binding domain, another region of the Tau protein, provides a large area for interacting with other cellular components, such as actin, kinesin, and dynein, thus ensuring the movement of cargo packages from the cytoplasm to the distal end of the axon [31, 32]. Over the last several years, a larger number of studies have revealed that the Tau protein is encoded by the MAPT gene and can interact with MTs in a “kiss-and-hop” fashion, namely, temporarily dwelling on a single MT before hopping to the adjacent MT, regulating MT dynamics [33, 34]. This dynamic MT Tau interaction is maintained mainly through electrostatic interactions between the positively charged MT-binding region and the negatively charged acidic glutamate-rich C-terminal regions of the tubulin surface [35, 36]. In addition to these electrostatic interactions, posttranslational modifications of Tau, mainly phosphorylation and acetylation, strongly affect MT-Tau binding and thereby have the potential to modulate the organization of MTs [37, 38].

DCX is a unique MAP that has been shown to be involved in MT assembly, turnover, and posttranslational modification of *α*- and *β*-tubulin proteins [39]. Binding of DCX to tubulin increases MT homeostasis in neurons, which can be disturbed via knockout of the DCX gene sequence using an inducible transgenic mouse approach [40]. Additionally, DCX is expressed in various regions of the developing nervous system and is regarded as a gold standard biomarker for identifying neuronal precursors and migration during adult neurogenesis [41, 42].

In addition, fibroblast growth factor 13 (FGF13) acts as an intracellular MAP that promotes axonal development, neuronal polarization and migration, and brain development [43]. It is rich in the central nervous system, especially for the developing brain. The regulatory mechanism by which FGF13 induces MT polymerization and stabilization is through binding to a tubulin-binding domain to directly interact with tubulin and colocalize with MTs in the growth cone [44]. Accumulating evidence suggests that overexpression of FGF13 enhances axonal regeneration and functional recovery by maintaining MT stabilization following spinal cord injury (SCI) and FGF13 deficiency causes cognitive impairment due to delayed neuron migration in both the cortex and hippocampus [44, 45]. Overall, these MT-stabilizing proteins are essential for cytoskeletal reorganization, growth orientation, and intracellular organization.

Several regulatory proteins can interact with dynamic MT minus-ends to catalyze the removal of tubulin subunits. These MT-destabilizing proteins are mainly MT depolymerases or the members of the calmodulin-regulated spectrin-associated protein (CAMSAP) family. MT depolymerases, also known as kinesin family proteins, have been reported to break the lateral links of protofilaments and tear off the tubulin monomer from the spindle by attaching to the minus-end of MT [46]. Currently, over 40 known kinesins have been identified in mammalian cells and are constitutively expressed in neurons [47]. Kinesins are known to modulate various cellular functions, including energy transport, spindle elongation during cell division, and alteration of the MT dynamics [48]. Some studies have indicated that kinesins that regulate neuronal behavior and function are closely associated with the MAPK cascade. For instance, kinesin-8 connects and interacts with the MAPK pathway to induce neuronal migration and differentiation [49]. Meanwhile, kinesin-5-induced MAPK signaling activation regulates myelination of the nervous system and promotes neuronal polarization and morphogenesis in cortical pyramidal neurons [50, 51]. Similar to the functions of the kinesin family, the CAMSAP family also binds to free minus-ends of MTs to slow tubulin addition, leading to the arrest of their growth at minus-ends [52]. This family of proteins, including CAMSAP1-3, contains an amino-terminal CH domain. In worms and mammals, CAMSAPs are localized to the outermost tips of the minus-ends, which play a crucial role in transporting new MTs into an axon or a dendrite in neuron differentiated from neural stem cells (NSCs) [53, 54]. A study by Cao et al. recently proposed that CAMSAP2 was capable of slowing minus-end polymerization and facilitating polarized cargo trafficking, which strengthened MT organization [55]. Additionally, Pongrakhananon et al. revealed that CAMSAP3 was required to retain a dynamic pool of MTs as it prevented *α*TAT1-mediated acetylation and thus maintained neuronal polarity [56]. Depletion of neuronal CAMSAP3 reduced dynamic MTs, resulting in supernumerary axon formation [56].

## 4. MT Organization in Neurons

A vertebrate neuron is an exquisitely polarized cell whose structure is composed of a cell body, a single elongated axon, and several dendrites [57]. In neuronal networks, axons play the major role in transmitting information and transporting macromolecules, while dendrites form numerous spine apparatuses for receiving information [58, 59]. During neuronal development, the cell body initially produces several short motile lamellipodia (stages 1-2); one of these lamellipodia rapidly becomes the axon (stage 3), while the remaining neurites transform into dendrites and gradually mature to build neural networks (stages 4–5) [60]. At stages 1-2, a fan-shaped structure is found at the tip of the growing axon; this growth cone can perceive the surrounding environment changes and regulates the rate and direction of axon extension, guiding axons over long distances to connect their specific targets [61]. If axons fail to grow due to a hostile local environment (e.g., hemorrhages, ischemia, the accumulation of inflammatory factors, myelin debris, or axonal inhibitory molecules), the tips of growing neurites form retraction bulbs. According to the cytoskeletal organization, the growth cone can be separated into three regions: peripheral (P-) and central (C-) regions as well as the transitional zone (T-zone). The P-region contains actin-rich lamellar protrusions, and its surface stretches out many lamellipodia and filopodia, which are pivotal to control the extension and retraction of the growth cone [62]. The C-region, located at the base of the growth cone, is the MT-rich region contiguous with the axonal shaft that shapes the morphology of the growth cone and orchestrates cytoskeletal remodeling. The T-zone is located between the P- and C-regions. Such domain encompasses actin arcs, a dense meshwork of actin filaments that creates a barrier to hinder MT forward from the C-region to the P-region [63, 64]. For the growth cone to advance, a dense MT array from the C-region must penetrate the T-zone to reach the P-region, preferentially polymerizing after incorporation of GTP to the *β*-tubulin subunit [65]. Axonal protrusion, retraction, and turning in response to signaling cascades require the coordination of the neuronal MT network and the actin cytoskeleton within the growth cone [66]. Specifically, recent studies have shown that growth cone steering and advancement in response to environmental cues depend on MT assembly and dynamics [67, 68]. Thus, understanding the intrinsic regulatory mechanisms of MT dynamics and function within the growth cone may provide therapeutic targets for interventions that improve axon growth and guidance during neurodevelopment and neuroregeneration following injury.

**Figure 2:**
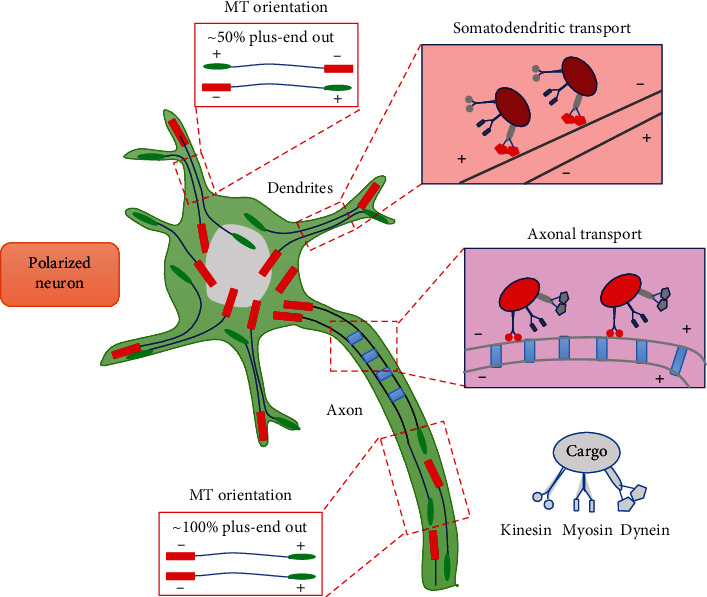
Basic mechanisms of MT organization during the differentiation of neurons into axons or dendrites. In axons, MTs display uniform polarity orientation with their plus-ends out. This array is essential to drive cargo transport through the proximal-end to the distal-end by kinesins, whereas in dendrites, MTs are mixed polarity with half of their minus-ends pointing to the soma, which allows dynein motors to selectively transport cargoes across this mixed MT arrays.

Neuronal MTs are arranged in a specific orientation [9, 69]. In axons, MTs have an almost exclusive plus-end-out orientation, whereas in dendrites, MTs have an antiparallel organization with equal proportions of plus-end and minus-end towards the soma [70]. This distinct orientation is partially regulated by kinesin and dynein, two molecular motor proteins that cooperate with protofilaments to drive MT elongation from the C-region to the P-region [71, 72]. Moreover, these proteins also act as vectors for transporting organelles and other cargo towards axons or dendrites [73]. Kinesins have been determined to move cargo to axon terminals, whereas MTs of mixed polarity allow dynein motors to drive cargoes, such as that from the Golgi apparatus and ribosomes, specifically into dendrites [74]. Such distinct MT polarity patterns and cargo sorting are essential for distinguishing neurons into axons or dendrites (Figure 2).

The overall appearance and arrangement of the MT network within neurons are variable and depend on their maturation stage [75, 76]. At the early stages of initial neurite outgrowth, MTs are of mixed polarity as short mobile polymers are rapidly released to be used by other MTs for their elongation. However, at the later stages of development, i.e., adult neurons, MTs have minus-end-out orientation with hundreds of micrometers in length and act as major long-distance railways for organelle transport. During development, MT orientation according to a network of feedback loops is essential for maintaining proper neuronal shape and inducing neuronal polarization [77, 78]. Upon neuronal polarization, posttranslational modifications of MTs in the nascent axon provide selective transport routes that increase the neurite length-dependent feedback and anterograde transport [79, 80]. The establishment of a normal feedback loop network is required for the activation of various signaling cascades and for modulation by several molecules [81, 82]. For instance, Shootin1 is a brain-specific cytoplasmic molecule that can be detected in the MT-associated protein fraction [83]. It is highly expressed in axonal growth cones and plays the central role for promoting neuronal polarity and axon outgrowth through a self-promoting feedback loop involving the Rac1/Pak1 signaling cascade [84, 85]. This facilitatory effect provides the driving force to induce one neurite under the growing state, while the remaining neurites are still in a pause state, ultimately driving neuron-autonomous neuronal polarization to generate a long signal-sending axon and several shorter signal-receiving dendrites.

## 5. MT Modifications in Nervous System Injury

Injury to the central nervous system (CNS) induces severe neurological complications for individuals with traumatic brain injury (TBI) or SCI because various inhibitory factors secreted by oligodendrocytes and scar-forming cells and the poor intrinsic growth ability upon neuronal maturation hamper axon regeneration and functional recovery [86, 87]. A growing number of studies have recently identified MTs as promising targets for coaxing regeneration of injured adult axons [88, 89]. The functions of MTs include (1) providing a structural backbone to maintain axon-specialized morphologies [57], (2) acting as the major long-distance railways for substrate transport in both directions [90], (3) ensuring the growth and steering of developing axons [91], and (4) regulating the extent of regeneration in injured axons [9]. Accordingly, moderate stabilization of MTs by Taxol enables MT polymerization and cytoskeleton organization, which transform the nongrowing retraction bulbs into growing axons [92]. Conversely, adding the MT-depolymerizing drug nocodazole to cultured dorsal root ganglia (DRG) neurons disturbs cytoskeleton organization and dynamics and increases the number of retraction bulbs [93].

TBI involves trauma to the CNS with characteristics of hypotension, hypoxia, and behavioral/cognitive abnormalities [94]. A common characteristic of TBI is loss of axonal integrity and cytoskeletal derangement, which are intrinsically associated with MT deficits and axonal dysfunction. Recently, MT disruption and loss, manifesting as MT depolymerization and the decrease of related proteins, such as Tau, p-Tau, and acetublin, were found to be key ultrastructural hallmarks of brain damage [95]. Thus, inducing MT stabilization is a novel therapeutic strategy to protect the damaged brain from high intracranial pressure and ischemia. A previous study reported that maintaining MT stabilization by local administration of FGF13 promoted neuronal migration and axon formation; in contrast, suppression of FGF13 expression delayed neuron migration and brain development [44]. Further studies revealed that administration of an MT-stabilizing drug, epothilone D, altered synaptic plasticity and dampened detrimental neuroglial responses after mild TBI of mice for 1 week [96]. However, intraperitoneal injections of low doses of exogenous nocodazole after TBI destroyed MT assembly and triggered a degenerative response characterized by loss of synapses and abnormal cytoskeletal rearrangement, as well as impairments in learning and memory [97]. While stabilizing the MT cytoskeleton is vital for ameliorating damage from TBI, there is still much to learn about the potential mechanisms involved in controlling this process.

SCI induces severe neurological deficits causing MT disorganization and deregulation of the MT cytoskeleton. Accumulating evidence has demonstrated that MT formation and stabilization are important for maintaining cytoskeletal integrity and axonal transport, as well as preventing detrimental gliotic responses [98]. Remodeling axonal MTs through genetic intervention or pharmacological treatment significantly enhanced axon regrowth and extension and reduced scar formation in an *in vivo* model of traumatic SCI, whereas the inhibition of MT stabilization by nocodazole weakens this beneficial effect [99]. Duan et al. demonstrated that systemic administration of epothilone B, an MT-stabilizing agent, reconstructed neovascularization by facilitating apoptosis and the migration of endothelial cells and pericytes and promoting their proliferation after SCI [100]. Moreover, regulation of MT dynamics by upregulating FGF13 expression is essential for promoting growth cone initiation, neuronal polarization, and regeneration of damaged axons following SCI [45]. Further studies found that MSAs also prevented fibroblast migration and prolonged the retention of MAPs, reducing inhibitory fibrotic scarring and improving intrinsic growth capacity, ultimately improving spinal cord restoration after injury [101–103]. In addition, recent studies on the relationship between autophagy and MT dynamics revealed that autophagy activation increased the expression of acetylated MT, a key modification for controlling MT stability and growth, thus attenuating axonal retraction and consequently enhancing locomotor recovery after SCI [104, 105]. Overall, stabilizing MTs in damaged neuron plays a pivotal role in determining their regenerative capacity after SCI.

## 6. MT-Targeting Agent

**Figure 3:**
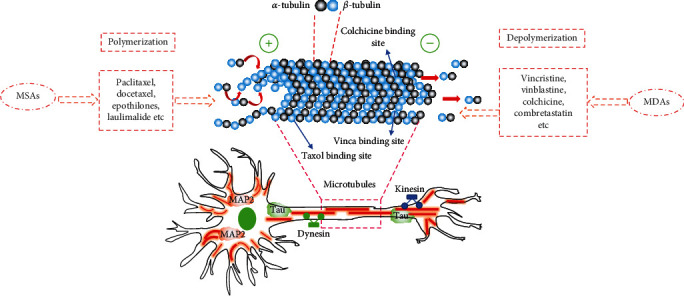
Diagram of MAPs, MSAs, and MDAs involved in the regulation of MT dynamics within neurons. MT organization and dynamics are regulated by MT proteins, MSAs and MDAs. MT-targeting agents can interfere with the dynamic equilibrium of MT polymerization and depolymerization. According to their mechanisms of action, MT-targeting agents can be divided into two groups: MSAs and MDAs. The former includes paclitaxel, docetaxel, epothilones, and laulimalide. They exert bind and interfere with the Taxol-binding domain and nontaxane sites. MDAs include vincristine, vinblastine, colchicine, and combretastatin. They depolymerize MTs by targeting the vinca-binding domain and colchicine-binding domain. These MT-targeting agents influence the polymerization and depolymerization of MTs and are patterned by a variety of MAPs, including MAP2, Tau, dynein, and kinesin. These MAPs play the critical roles in mediating a plethora of cellular processes such as cell division and motility, intracellular transport, axonal specification, and neuronal development.

MT-targeting agents can be classified into two main categories: MSAs and MDAs. The former, including paclitaxel, docetaxel, epothilones, and laulimalide, can bind to the tubulin heterodimer at the plus-end to promote the polymerization of tubulin to MTs. The latter induces MT dysfunction at the end of the mitotic spindle by preventing tubulin polymerization on both plus- and minus-ends of MT, leading to the arrest of mitosis. Representative examples of MDAs include vincristine, vinblastine, colchicine, and combretastatin. The regulatory mechanism by which MT-targeting agents influence MT dynamics depends on which MT domain they bind to [106] (Figure 3). According to their binding affinities to tubulin, these binding domains can be categorized as Taxol-binding domain, colchicine-binding domain, and vinca-binding domain [107]. MSAs are able to target the cytoskeleton and inhibit cell division by binding to *β*-tubulin of inner surface of MT lumen, which is generally described as the Taxol-binding domain [108]. Taxol-binding drugs, such as laulimalide, are shown to increase MT polymerization and assembly by allosterically stabilizing the Taxol-site M-loop [109]. MDAs depolymerize MTs by interacting with the vinca-binding domain or colchicine binding domain. Vincristine and vinblastine are two typical vinca-binding analogues. They induce mitotic arrest and block cell division by blocking tubulin polymerization at the interdimer interface, which is named the vinca-binding domain [110]. Colchicine and combretastatin are colchicine-binding analogues. They bind to the Cys241 residue of *β*-tubulin (termed colchicine-binding domain) via hydrogen bonding to induce mitotic arrest and chromosome missegregation [111].

MT-targeting agents were traditionally used as anticancer agents for the treatment of various solid tumors [112]. To date, some of these agents are commonly approved for clinical anticancer chemotherapy for many types of solid tumors. However, recent work has shed light on their potential for treating traumatic nerve damage and neurodegenerative diseases, including Alzheimer's disease (AD), Parkinson's disease (PD), amyotrophic lateral sclerosis (ALS), and SCI (Table 1). This dual treatment ability aroused our curiosity. MTs are found in all characterized eukaryotic organisms, but exert diverse cellular functions in different cell types [113]. In cancer cells, MTs are one of the important components of the mitotic spindle and are capable of pulling sister chromatids towards opposite poles [114]. Thus, MT assembly and disassembly are critical for determining the proliferative capability of cancer cells. As mentioned in the previous passage, MT-targeting agents can interfere with the corresponding binding domains of tubulin to block their polymerization. This event impairs the ability of spindle MTs to capture chromosomes and interferes with the G2/M phase of the cell division, leading to mitotic arrest and even death in cancer cells [115]. In neurons, MTs, as one of the major longitudinal cytoskeletal filaments, are abundant in axons and dendrites [116]. Additionally, adult neurons or permanent differentiated neurons lack high proliferative capacity, resulting in few concerns and studies that are focused on MT-regulating neuronal division after damage or degeneration. Based on this fact, current studies are concentrated on the role of MT stability in regulating axon growth and steering as well as the intracellular trafficking of cargos during neuronal development [9, 116]. Following neuronal injury or neurodegenerative diseases, MTs become disassembled and gradually lose mass, leading to axonal atrophy and degeneration [117]. MSAs, such as Taxol and epothilones, promote the polymerization of tubulin to MTs during disease and/or injury [106, 118]. Thus, MSAs are regarded as potential candidates for treating neuronal disorders. However, the precise mechanism of MSAs for treating cancer and neuronal diseases needs to be explored in future research. In this section, we will introduce some background information on these therapeutic compounds and elucidate their application.

MSAs have been used as cancer therapeutic drugs for more than 20 years [130]. They were originally derived from natural resources. For instance, paclitaxel (Taxol®) was the first MSA to be isolated (derived from the Pacific yew tree in 1971) and approved by the FDA for the treatment of breast cancer, with an optimal therapeutic concentration of 260 mg/m^2^ [131]. This is decomposed into 6*α*-hydroxy-paclitaxel by activating CYP2C8 enzyme in human liver microsomes [132]. Due to the difficulty of obtaining paclitaxel from the plant, various analogues, such as docetaxel, cabazitaxel, larotaxel, and TPI-287, have been synthesized through modification of its side chains and have exhibited encouraging clinical efficacy for treating breast cancer [133, 134]. Recent studies have shown that paclitaxel can maintain specialized neuronal morphology and support axonal and dendritic transport by resisting MT dynamic instability. For instance, Hellal et al. found that stabilizing the MT network with Taxol hindered the formation of scarring and prevented axonal retraction and swelling after SCI in rodents [119]. In another work, paclitaxel encapsulated in a collagen microchannel was shown to enhance neuronal differentiation of NSCs *in vitro* and improve axonal transport and axonal functional recovery in a complete spinal transsection injured models of rats [135].

Epothilones are another class of MSAs that include a 16-membered macrocyclic lipid compound [136]. To date, six natural epothilone variants, i.e., epothilones A-F, can be easily obtained at a large scale by isolation from soil bacteria or by chemical synthesis [137]. Compared with paclitaxel, epothilones are more soluble in water and have better tumor resistance, which make them a viable alternative to paclitaxel in facilitating antitumor activity [138]. Data from phase III clinical trials revealed that ixabepilone, a semisynthetic analogue of epothilone B, effectively improved the survival rate of patients with metastatic breast cancer to 70% with an intravenous infusion dose of 30 mg/m^2^ every 3 weeks [139]. In addition to treating cancer, a series of studies have shown that epothilones possess therapeutic potential for repairing neurological disorders. For instance, in a Tau transgenic mouse model of tauopathies characteristic of AD, intraperitoneal injections of 3 mg kg^−1^ epothilone D once weekly for a 3-month period were demonstrated to support MT assembly and axon extension as well as reduce brain cognitive deficits [140]. In addition, this compound also exhibited beneficial effects on neuronal differentiation of cultured NSCs *in vitro* and improved axonal sprouting and functional recovery *in vivo* following SCI [103, 141]. Additionally, epothilones are easier to administer than paclitaxel due to their higher water solubility, which endows epothilones with the capability to cross the blood-brain barrier [138]. In short, compared to paclitaxel, epothilones show several physicochemical advantages including (1) increased water solubility for direct delivery without solvent, (2) a lack of intracellular toxicity and strong antineoplastic activity, and (3) the capacity of easily crossing the blood-brain barrier. In addition, the chemical structure of epothilones exhibits 16-membered macrocyclic lactones, which can produce synthetic analogues during clinical drug design [142]. It has been shown that systemic administration of epothilone B moderates MT stabilization to reduce fibrotic scarring and increase axon growth after SCI [102]. Additionally, epothilones augmented axonal growth and improved skilled limb function after cortical stroke in the brain [143]. Thus, both paclitaxel and epothilones are regarded as attractive therapeutic compounds for promoting the functional and structural recovery from neurodegenerative diseases and disorders.

MDAs, also called polymerizing inhibitors, possess the ability to promote the depolymerization of MTs by interfering with the colchicine-binding domain and vinca-binding domain to block cell division and interfere with the formation of a normal mitotic spindle. Thus, they have shown a strong antiangiogenic and antivascular activity and offer a pharmaceutical opportunity for treating different tumor types, including breast, lung, ovarian, and hematologic tumors [144]. According to their tubulin-binding domains, MDAs can be further divided into two groups: vinca-binding analogues and colchicine-binding analogues [145]. The representative example in the former is vinca alkaloid (VA), a natural chemotherapy agent obtained from the Madagascar periwinkle plant in 1950. It was first approved by the FDA for the clinical treatment of lung cancer and breast cancer, but provoked severe neurotoxicity [146]. To overcome this defect, other VA analogues, including natural (vinblastine and vincristine) and semisynthetic (vinorelbine, vindesine, and vinflunine) analogues, have gained attracted attention in cancer treatment [147]. Data on clinical pharmacokinetics revealed that the terminal half-lives of VA and its derivatives range from 18 to 85 h [148]. Furthermore, they were found to be first metabolized within the liver through the action of cytochrome P450 CYP3A4 and then subjected to biliary elimination and finally excreted into the feces [148, 149]. It should be addressed that VA and its derivatives can rapidly enter the peripheral tissues, including the peripheral nervous system; thus, administration of these antimitotic agents probably causes characteristic peripheral neurotoxicity [150]. To overcome this defect, researchers have identified three beneficial strategies to reduce these adverse effects, i.e., combination with other drugs, investigation of novel drug delivery platforms, and the synthesis of new VA analogues [147].

Colchicine, an alkaloid derived from the meadow saffron plant, belongs to the group of colchicine-binding analogues and is used as a therapeutic drug for anticancer treatment, such as lung, breast, and gynecological cancers [151]. Similar to VA metabolism, colchicine is broken down by the CYP3A4 enzyme within liver microsomes [152]. However, colchicine may cause toxicity in normal cell proliferation. A phase II clinical trial established that the safe dose of colchicine was 0.015 mg kg^−1^, a higher dose of 0.1 mg kg^−1^ resulted in intoxication, and the maximum fatal dose was 0.8 mg kg^−1^ [153–155]. Due to colchicine's low therapeutic index and severe cytotoxicity, various colchicine analogues have been synthesized by modifying the tricyclic-membered rings. Urbaniak et al. synthesized 16 novel colchicine derivatives, namely, double- (4-7) or triple-modified (17-28) urethanes, and found that these novel colchicine derivatives (IC_50_ range of 1.1-6.4 nM) had higher antiproliferative activity than colchicine (IC_50_ = 8.6 nM) by testing the viability of primary breast cancer cells [156]. Recently, the colchicine analogue combretastatin was found to promote anticancer activity by inhibiting the elongation of MTs [157]. Moreover, its synthetic derivatives show strong antioxidant activity and anti-inflammatory activity. For instance, Huang et al. demonstrated that the combretastatin derivatives NTU-228 and HK-72 exhibited significant leukocyte inflammatory responses by quantifying N-formyl-Met-Leu-Phe- (fMLF-) induced reactive oxygen species production in human leukocytes [158]. Evidence from antioxidant studies in a subcutaneous dorsal CaNT tumor model revealed that combretastatin A-4, a combretastatin analogue, had strong protective effects against hydroxyl radicals and radical-based DNA damage [159].

## 7. Concluding Remarks and Future Perspectives

As briefly summarized here, MTs are dynamic cytoskeletal filaments that carry out the distinct functions of cellular physiology, such as cell division and motility, organelle positioning, and intracellular transport. Generally, MTs exhibit extensive dynamic instability, i.e., constant transition between phases of growing and shrinking. This allows them to enhance tubulin–tubulin interactions, create pushing and pulling forces, and direct cell locomotion via crosstalk with the actin cytoskeleton [160]. *In vitro*, the dynamic state of MTs can be recorded by measuring their rate of growth and shrinkage or by quantifying their mass [161]. The most common and reliable way to track MT dynamics is using high-resolution imaging methods, including fluorescent speckle microscopy and cryo-electron microscopy [162]. Additionally, MT acetylation is a common posttranslational modification that can protect MTs against mechanical stresses by polarizing the centrosome or the mitotic spindle within the cell [163]. Eliminating MT acetylation by reducing the activity of *α*-tubulin acetyltransferase 1 enzyme does not impair protofilament organization but does cause a reduction in MT dynamics [164]. In brief, understanding the role of MT dynamics in cargo transportation and cytoskeletal reorganization has provided further insights into the biophysics and biochemistry of MT function involved in neuronal development, disease, and injury.

Given the importance of MT dynamics in cytoskeleton reconstruction, designing novel compounds that facilitate MT polymerization or upregulate MT acetylation has received increasing attention, due to the applications for different neurotraumatic diseases [57]. Encouragingly, some works have achieved positive therapeutic effects in cellular and animal models. For instance, systematic administration of epothilone B, a kind of MT-stabilizing agent, not only facilitated the growth of both sensory and motor axons in an unfavorable environment *in vitro* but also demonstrated dual effects on improving intrinsic neuron growth and reducing fibrotic scar in an *in vivo* SCI model [101, 102, 165]. While some natural MSAs can cause significant adverse side effects, including neutropenia, chemotherapy-induced peripheral neuropathy, and alopecia when used in cancer treatment at a high concentration [106, 166], researchers have proposed alternative and beneficial strategies, including synthesis of new MSA analogues, design of novel drug delivery platforms, and combinations with other drugs, to reduce these adverse effects [147]. Additionally, application of these therapeutic strategies can reduce the dosage of MSAs and their analogues; thus, administration at low concentrations can significantly enhance axonal regeneration and functional recovery in neuronal injury models. Further studies addressing the mechanisms that regulate the dynamic remodeling of MT networks will greatly increase our understanding of the intricacies of MT organization in various cell types.

## Figures and Tables

**Table 1:** Summary of various MT-targeting agents applied to protect the nervous system.

Classification	Compound	Pathological model	Outcome	Ref.
MSAs	Paclitaxel (Taxol)	SCI	Enhancement of nerve regeneration and functional recovery	[119, 120]
		Retinal nerve injury	Increased MT numbers and stabilization to restore axonal transport	[121]
		AD	Improvements in axonal transport, tissue, and motor function	[122]
	Epothilones	SCI	Decreased scarring, increased axon regeneration, and improved motor function	[102, 103]
		PD	Rescued MT defects and attenuated nigrostriatal degeneration	[123]
		AD	Reduced axonal dystrophy, increased axonal MT density, improved speed of axonal transport, and improved cognitive performance	[124, 125]
	Davunetide (NAP)	AD, ALS	Prevented axonal transport disruption, synaptic defects, and behavioral impairments	[126, 127]

MDAs	Vincristine	iPSC-derived neurons from HSP patients	Ameliorated axonal swelling	[128]
	Okadaic acid	Hyperphosphorylated Tau to model AD	Reduced the growth of the rat cortical neuron axons	[129]

## Data Availability

No new data are generated in this study.

## Abbreviations

MT: Microtubule
MTs: Microtubules
MAPs: Microtubule-associated proteins
MTAs: Microtubule-targeting agents
*γ*-TuRC: The gamma-tubulin ring complex
GCPs: Gamma-tubulin complex proteins
MZT1: Mitotic spindle organizing protein 1
CM1: N-terminal centrosomin motif 1
MTOCs: Microtubule organizing centers
MAP: Microtubule-associated protein
DCX: Doublecortin
FGF13: Fibroblast growth factor 13
SCI: Spinal cord injury
CAMSAP: The calmodulin-regulated spectrin-associated protein
MSAs: Microtubule-stabilizing agents
MDAs: Microtubule-destabilizing agents
NSCs: Neural stem cells
CNS: The central nervous system
TBI: Traumatic brain injury
DRG: Dorsal root ganglia
AD: Alzheimer's disease
PD: Parkinson's disease
ALS: Amyotrophic lateral sclerosis
VA: Vinca alkaloid.
